# HIV-1 capsid variability: viral exploitation and evasion of capsid-binding molecules

**DOI:** 10.1186/s12977-021-00577-x

**Published:** 2021-10-26

**Authors:** Akatsuki Saito, Masahiro Yamashita

**Affiliations:** 1 Department of Veterinary Medicine, Faculty of Agriculture, University of Miyazaki, Miyazaki, Miyazaki Japan; 2 Center for Animal Disease Control, University of Miyazaki, Miyazaki, Miyazaki Japan; 3 Aaron Diamond AIDS Research Center, Columbia University Vagelos College of Physicians and Surgeons, New York, NY USA

**Keywords:** HIV-1, Lentiviruses, Capsid, Host factors, Inhibitors, Sequence variation

## Abstract

The HIV-1 capsid, a conical shell encasing viral nucleoprotein complexes, is involved in multiple post-entry processes during viral replication. Many host factors can directly bind to the HIV-1 capsid protein (CA) and either promote or prevent HIV-1 infection. The viral capsid is currently being explored as a novel target for therapeutic interventions. In the past few decades, significant progress has been made in our understanding of the capsid–host interactions and mechanisms of action of capsid-targeting antivirals. At the same time, a large number of different viral capsids, which derive from many HIV-1 mutants, naturally occurring variants, or diverse lentiviruses, have been characterized for their interactions with capsid-binding molecules in great detail utilizing various experimental techniques. This review provides an overview of how sequence variation in CA influences phenotypic properties of HIV-1. We will focus on sequence differences that alter capsid–host interactions and give a brief account of drug resistant mutations in CA and their mutational effects on viral phenotypes. Increased knowledge of the sequence-function relationship of CA helps us deepen our understanding of the adaptive potential of the viral capsid.

## Introduction

The HIV-1 capsid is a conical shell that encases viral nucleoprotein complexes necessary for replication [1]. The capsid protein (CA), the major building unit of the viral capsid, is part of the precursor Gag protein in immature particles and liberated upon Gag cleavage to form mature capsids. CA–CA interactions play a critical role during virion morphogenesis [2, 3]. The mature capsid, which is composed of around 250 CA hexamers and 12 CA pentamers, orchestrates multiple post-entry processes, including reverse transcription, nuclear entry and integration targeting [1, 4–6]. These capsid-mediated events depend on capsid disassembly, a highly coordinated process that needs to be executed in a temporally and spatially controlled manner for optimal infectivity [7, 8]. The HIV-1 capsid directly interacts with multiple host factors that can either promote or thwart virus infection [9]. The complex interplay between HIV-1 capsid and cellular factors defines the fate of incoming virus particles in target cells.

Over the last two decades, an increasing number of cellular factors that bind to the HIV-1 capsid have been discovered and extensively studied in detail [9–11]. In the meantime, a number of small-molecule compounds were developed that target the capsid and block HIV-1 infection with different mechanisms of action [12, 13]. Previous studies aimed at investigating the consequence of capsid interactions with these molecules have advanced our understanding of the fundamental principles of capsid-mediated processes during HIV-1 replication. These studies employed a variety of techniques from multiple disciplines and assessed a diverse set of lentiviral capsids. These efforts have led to the accumulation of a large amount of data on the sequence-fitness landscape of the HIV-1 capsid, and provided the detailed knowledge of phenotypic outcomes caused by mutations or sequence variation in CA.

**Fig. 1:**
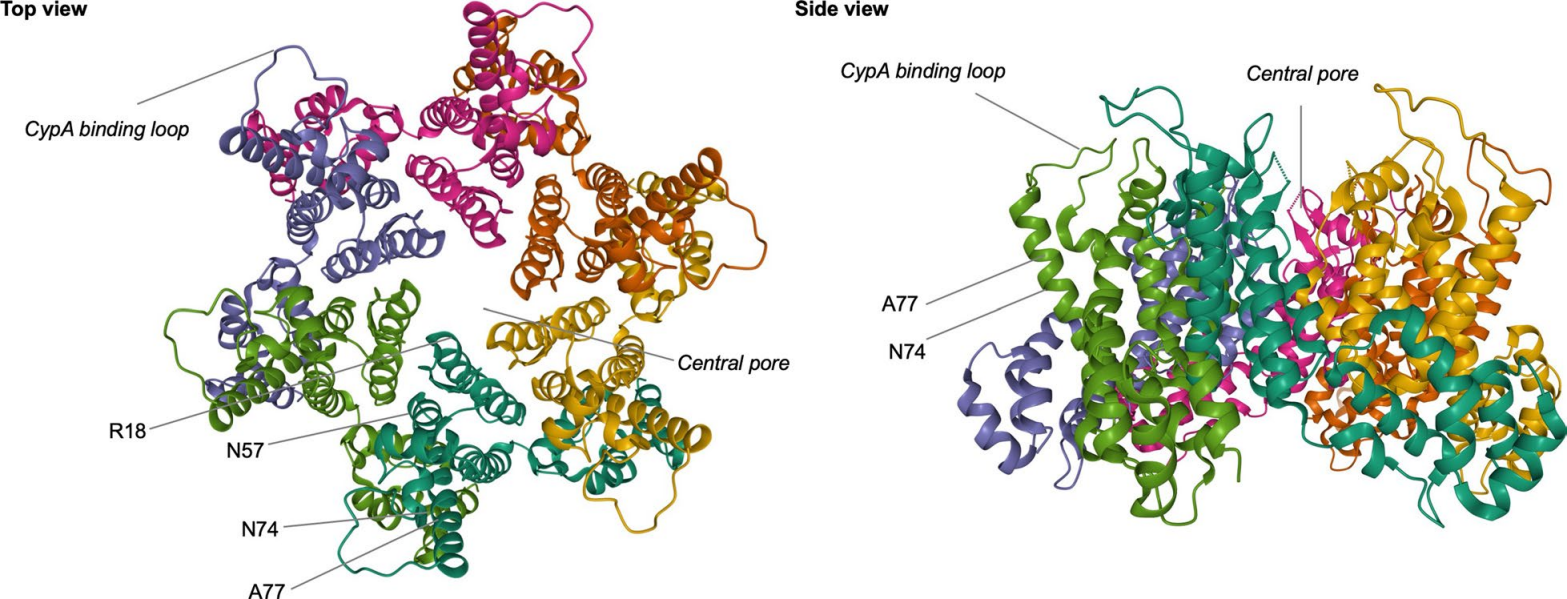
Host factor interaction sites in the HIV-1 CA hexamer. Structure of an HIV-1 CA hexamer (PDB: 4XFY) is shown in a top (left) or side view (right) with each protomer depicted in a different color. Amino acid residues or regions that mediate interactions with major host factors are indicated

This review article provides an overview of the phenotypic consequences of sequence variation in CA. Naturally occurring polymorphisms and lab-derived mutations are two major sources of CA sequence variation (summarized in Table 1; Fig. 1). We will focus on the results from recent literature exploring CA sequence variations that modulate HIV-1 binding to capsid-binding molecules and ultimately affect phenotypic properties of HIV-1 (Table 2). Lessons learned from these studies illuminate how capsid interactions with host factors shape HIV-1 biology, and also have implications for the evolutionary potential of the HIV-1 capsid, a property that is imperative to the emergence of drug resistance.

**Table 1:** List of well characterized capsid mutants

CA mutation	Phenotype	Location	References
Q4R	Decreased CPSF6 binding Renders an IFN-hypersensitive virus resistant to IFN-β Accelerates both reverse transcription and uncoating	β-hairpin	[139, 221]
R18G	Defects in reverse transcription and infectivity Loss of IP6 binding	Helix 1	[24]
P38A	Hypostable core	Helix 2	[22, 61]
E45A	Hyperstable core Cell cycle dependency	Helix 2	[22, 61]
N57A	Loss of CPSF6 binding Loss of PF74 sensitivity Cell cycle dependency	Helix 3	[22]
Q63A/Q67A	Hypostable core Cell cycle dependency	Helix 4	[22, 61]
N74D	Loss of CPSF6 binding Replication defect in macrophages MX2 resistance Integrates into gene-poor regions Utilization of non-canonical nuclear entry pathway Higher sensitivity to IFN-α Stimulates a type I IFN response in macrophages Sensitive to restriction by TRIM34	Helix 4	[93]
A77V	Loss of CPSF6 binding Integrates into gene-poor regions	Helix 4	[199]
H87Q	Naturally occurring polymorphism Reduced CypA binding Renders HIV-1 independent from CypA MX2 resistance	Cyclophilin A binding loop	[113]
G89V	Loss of CypA binding MX2 resistance Higher sensitivity to IFN-α	Cyclophilin A binding loop	[113]
P90A	Loss of CypA binding Higher sensitivity to human TRIM5α MX2 resistance Stimulates type I IFN response in primary macrophages	Cyclophilin A binding loop	[110]
A92E	CsA dependency Cell cycle dependency	Cyclophilin A binding loop	[140]
G94D	CsA dependency Cell cycle dependency	Cyclophilin A binding loop	[140]
G116A	Naturally occurring polymorphism Increases infectivity in simian cells MX2 resistance	Helix 6	[240]
R132K/L136M	CTL escape mutations CsA dependency Cell cycle dependency	Helix 7	[146]
R143A	Resistance to TRIM-NUP153 Unable to stimulate cGAS-dependent innate immune activation in THP-1 cells	Helix 7	[22, 61]
K182R	Reduced CPSF6 binding	Helix 9	[188, 189]
Q219A	Hypostable core Cell cycle dependency	Helix 11	[22, 61]

**Table 2:** Capsid-dependent cellular proteins

Gene name	Gene ID	Also known as	Gene description	Strategy for gene discovery	References
CPSF6	11052	Cleavage and polyadenylation specific factor 6	Subunit of a cleavage factor required for 3′ RNA cleavage and polyadenylation processing	Expression cloning	[93]
Cyclophilin A	5478	PPIA	Member of the peptidyl-prolyl cis–trans isomerase (PPIase) family	GAL4 two-hybrid screen	[104]
FEZ1	9638	Fasciculat ion and elongation protein zeta 1	Ortholog of the *Caenorhabditis elegans* unc-76 gene that is necessary for axonal bundling and elongation within axon bundles	RNAi-mediated inhibition	[100]
KIF5B	3799	Kinesin family member 5b	Member of the kinesin family protein with ATPase that acts as a microtubule motor	Pharmacological and RNAi-mediated inhibition	[125]
MX2	4600	MXB, MX dynamin like GTPase	Member of both the dynamin family and the family of large GTPases	Transcriptional profiling	[156, 292, 293]
NUP153	9972	Nucleoporin 153	Nucleoporin carrying characteristic XFXFG pentapeptides	Large-scale RNAi screening	[356, 357]
NUP358	5903	RANBP2	A large RAN-binding protein that localizes to the nuclear pore complex	Large-scale RNAi screening	[356, 357]
SUN2	25777	Sad1 and unc84 domain containing 2	An inner nuclear membrane protein with a major role in nuclear-cytoplasmic connection	Sun2-null mouse cells	[129, 358]
TNPO3	23534	Transportin (TRN) 3/TRN-SR2	Nuclear transport receptor for serine/arginine-rich proteins	Yeast-two-hybrid and siRNA screens	[356, 357, 359]
TRIM5α	85363	Tripartite motif containing 5α	Member of the tripartite motif (TRIM) family	Expression cloning	[230]
TRIM34	53840	Tripartite motif containing 34	Member of the TRIM family	HIV-CRISPR screening	[290]

## Assembly and maturation

Virion morphogenesis, a key process for HIV-1 replication, depends on the proper assembly and maturation of virus particles [3, 14, 15]. Mutagenesis studies showed that many amino acid changes in the CA domain of Gag block infectious virus production. Some mutations in CA inhibit release of virus particles, while other mutations produce particles that are not infectious. Viral assembly and maturation processes are blocked by perturbation of either CA–CA interactions or CA interactions with host factors.

### Immature particle assembly

The CA domain of Gag facilitates the formation of immature virus particles by providing critical protein–protein contacts for Gag multimerization [16, 17]. Unbiased and structure-guided mutagenesis studies carried out in the past identified a multitude of CA mutants that are blocked at virus assembly [18–24]. The vast majority of these mutations reside in interfaces in the CA domain that participate in Gag multimerization [3, 15]. The CA C-terminal domain (CTD) contains three well characterized sequence elements that promote Gag assembly. Dimeric interactions of CA subunits between neighboring hexamers are mediated by interfaces containing two hydrophobic residues (W184 and M185) in the helix 9 of CA [22, 25]. The carboxy-terminal end of CA and beginning of the SP1 region forms a six-helix bundle to promote Gag multimerization [26–30]. A third sequence element is the major homology region (MHR), a stretch of 20 amino acids highly conserved among diverse retroviruses, which is important for Gag hexamerization [31–35]. The CA N-terminal domain (NTD) also participates in Gag–Gag interactions [22, 36].

A major phenotypic change caused by many CA mutations at the interfaces critical for Gag multimerization is the reduction in particle production and release, which are assessed by measuring the quantity of Gag released in the supernatant or that of reverse transcriptase incorporated into particles. Several CA mutations were shown to prevent the formation of an immature structure using in vitro assembly assays [37, 38]. Additionally, some mutants defective for virus production impair Gag multimerization [39]. For instance, viruses carrying single mutations at the C-terminus of CA-CTD fail to form large Gag complexes as efficiently as the WT virus [28, 30]. Using centrifugation-based assays that can assess biophysical characteristics of assembly intermediates, different CA mutants were shown to be arrested at different stages during the assembly process in virus-producing cells [35, 40].

Interactions of the CA domain with host factors are also critical for immature particle assembly [41]. One such factor that has recently received considerable attention is inositol hexakisphosphate (IP6), which is a negatively charged metabolite [38, 42–44]. HIV-1 binding to IP6 during virus assembly is mediated by two positively charged lysine rings (K158 and K227 in CA amino acid numbering) located in the CA-CTD [45]. Amino acid substitutions at these two positions, such as K158A/I and K227A/I, impair viral infectivity by reducing particle production [45–48].

The requirement of IP6 for immature virus assembly has been studied for other lentiviruses. The two key lysine residues (K158 and K227) are both conserved in CA sequences from diverse lentiviruses. Production of distantly related HIV-1 Group O, HIV-2, and simian immunodeficiency virus (SIV) was reduced when IP6 was depleted by gene knockout of the inositol-polyphosphate multikinase (IPMK) gene or the inositol-pentakisphosphate 2-kinase (IPPK) gene, which regulates IP6 synthesis [46, 49]. In contrast, the dependence of non-primate lentiviruses on IP6 appears to be different. Knockout of the IPMK or IPPK gene reduced production of ferine immunodeficiency virus (FIV) particles [46]. Another study also showed a threefold reduction in production of FIV and equine infectious anemia virus (EIAV) in IPPK-deleted cells; however, re-introduction of IPPK did not affect virus production of both FIV and EIAV [49]. Additionally, both EIAV and FIV required a larger amount of IP6 than HIV-1 in in vitro Gag assembly assays [50]. Based on these observations, Ricana et al. have suggested that IP6 is required by primate lentiviruses for virion production and acts as an enhancer to virus particle release of non-primate lentiviruses [49].

### Mature capsid formation

Mature capsid formation is essential for production of infectious virus particles [16, 17]. Many CA mutants are capable of releasing virus particles, but display a lethal phenotype caused by a defect in the maturation process. During maturation, CA subunits liberated from the precursor Gag protein assemble to form mature cores that are competent for all the post-entry processes [51, 52]. The formation of mature cores is dependent on multiple CA–CA contacts, including intra-hexameric NTD–NTD and NTD-CTD contacts as well as CTD–CTD interactions that connect adjacent hexamers or pentamers [52]. A large number of mutations in these CA–CA interaction interfaces block mature capsid formation [22, 24, 53–58]. Many, but not all, CA mutants that are defective in maturation display aberrant core morphology; some particles lack a defined core, while others contain anomalously shaped cores [21, 22, 54–56, 59].

In some instances, HIV-1 CA mutants are capable of producing regular conical cores, which display size distributions similar to those of the WT virus, but their infectivity is severely impaired. One common mechanism of such phenotype is that CA mutations alter capsid stability. The requirement of optimal capsid stability has been demonstrated for replication of HIV-1, SIV, and Rous sarcoma virus (RSV) [55, 59–65]. Capsid stability and its effect on capsid disassembly have been studied in cell-free and cell-based assays, which will be described below.

A well-used cell-free experimental approach to measure capsid stability depends on biochemical assays in which viral cores are physically separated from detergent-treated virus particles using ultracentrifugation [61]. Core yield is frequently used as an indicator of capsid stability. This approach has been complemented by cell-free imaging assays [66–68]. In these microscopy-based assays, virus particles on the slide are permeabilized to promote the release of CA subunits before measuring the amount of CA that remains associated with virus particles. In this type of imaging assays, a virus carrying an E45A substitution, which is one of the first identified mutations that increase capsid stability, displayed a slower kinetics of CA release, whereas CA signals were quickly lost from virus particles of the hypostable K203A mutant [66–68]. These observations are consistent with those from the biochemical approach [61].

A group of capsid stability mutants described by Forshey et al. [22, 61] have been examined by multiple laboratories using a variety of cell-based assays to understand how intrinsic capsid stability influences capsid disassembly in virus-infected cells. There are generally three approaches to study capsid disassembly in cells [8]. Imaging assays visualize intracellular complexes and assess the state of uncoating by monitoring different readouts, such as CA molecules associated with cores, viral RNA exposure, and leakage of fluid markers [69–74]. Another approach involves the isolation of subviral complexes from virus-infected cells and their biophysical and biochemical characterization [75–77]. Finally, infectivity-based assays take advantage of a unique property of TRIM5 proteins, such as TRIM5α and TRIMCyp [78, 79]. The premise of TRIM5-based assays is that TRIM5 proteins only recognize the intact capsid and hence inhibit infection of viral particles that remain “coated”. In the cyclosporine A (CsA) washout assay, the drug CsA can be utilized as an off switch for TRIMCyp restriction by preventing its interaction with the viral capsid. A similar infectivity-based assay depends on the fact that virus-like particles, when added *in trans*, can saturate restriction of HIV-1 by TRIM5 proteins [78].

Overall, the results obtained with these assays showed that capsid stability mutations drastically alter the intracellular behavior of HIV-1 particles. In addition, intrinsic capsid stability generally correlates with intracellular capsid disassembly, albeit with a few exceptions. E45A and E128A/R132A mutations generate hyperstable cores [61] and delay the kinetics of capsid disassembly in cells [66, 71, 78, 80–85]. On the other hand, K203A and Q219A mutations destabilize cores [61], and the capsid disassembly of these mutants in target cells is accelerated [66, 69, 71, 83, 85, 86]. The P38A mutant is another example of the mutants with unstable cores [61]. The P38A mutant failed to enhance infectivity of reporter virus as efficiently as WT particles in TRIM5-based abrogation experiments [86, 87], while the same P38A substitution led to the loss of CA and the viral integrase protein in core-containing fractions in a fate-of-capsid assay [85].

On the contrary, more complicating phenotypes were observed for other mutants that moderately reduce capsid stability. The Q63A/Q67A mutant has unstable cores and uncoats faster than the WT virus in a cell-free assay [61]. However, in cell-based assays, Q63A/Q67A cores disassemble at a slower kinetics than WT cores [69, 81, 88, 89]. An R143A mutation displays varying phenotypes across different assays. In a cell-free assay, the R143A mutation decreased the core yield, but paradoxically it delayed the uncoating kinetics [61]. The R143A phenotype was equally variable in cell-based uncoating assay where the R143A mutant showed accelerated or delayed uncoating phenotypes, depending on the assays employed [69, 78, 90]. The discrepancy between cell-free and cell-based assays may be explained by effects of cellular factors on capsid disassembly in target cells. It is unclear what variables underlie the opposing phenotypes of the R143A mutant in different cell-based assays for capsid uncoating.

Capsid stability mutants vary in other post-entry processes. A block in infection of most of these mutants occurs at reverse transcription, regardless of the type of changes in capsid stability [21, 54, 56, 61, 91]. In contrast, a capsid stability mutant, Q63A/Q67A, is competent for reverse transcription, pointing to a block at nuclear entry [88]. In fact, several studies showed evidence for unusual phenotypes in nuclear entry caused by altered capsid stability. For instance, E128A/R132A and K203A mutants block nuclear envelope association and nuclear transport of pre-integration complexes (PICs) labeled with the APOBEC3F-YFP marker [70, 92]. Elevated accumulation of viral cores in the cytoplasm was observed for the hyperstable E45A mutant [83]. Furthermore, infection by many of these capsid stability mutants does not depend on nuclear entry cofactors exploited by many HIV-1 strains. E45A, P38A and Q63A/Q67A mutants were partially or completely insensitive to NUP153 knockdown [93–95].

IP6, a critical factor for immature particle assembly (see above), has been implicated in capsid maturation and stability [44, 45, 96–98]. A current model is that IP6 incorporated into HIV-1 particles is liberated after the protease-mediated cleavage of Gag and binds to an electropositive charged pocket created by arginine residues at position 18 (R18) of CA [42, 96, 99]. Interestingly, this pocket appears to be also utilized by the kinesin-1 adaptor protein FEZ1 [100, 101]. Lysine residues at position 25 (K25) were also shown to be involved in the IP6-dependent capsid maturation and dNTP import through dynamic capsid pores [68, 99, 102]. Mutations of R18 and K25 have moderate to severe defects in viral infectivity, which are accompanied by a reduced level of reverse transcription products [24, 42, 99]. IP6 binding differs between these mutants; R18G, but not K25N/A, lost IP6 binding, although both R18 and K25 mutants became insensitive to IP6 in cell-free assays for capsid stability and reverse transcription [99]. Virus particle morphology also differs between these mutants. R18G, K25I and K25N mutants do not alter virion morphology [24, 68]. In contrast, R18A is responsible for the altered capsid assembly property by the double mutant R18A/N21A, which forms a variety of abnormally shaped capsids [22, 58, 103], while most particles of the K25A mutant are defective [99]. However, all these mutants differ from the WT virus in their capsid assembly phenotypes when examined using recombinant proteins in vitro [42, 99]. Viruses bearing R18 or K25 substitutions display destabilizing effects on HIV-1 cores [61, 68, 99]. This finding fits well with the inability of K25A to abrogate TRIM5α restriction [99]. Importantly, a similar core-destabilizing effect was observed for K227 mutations, which reduce the amount of IP6 incorporated into virus particles [46]. Diverse lentiviruses contain positively charged amino acid residues (arginine or lysine) at the position corresponding to R18 in HIV-1, whereas the lysine ring does not seem to be conserved across primate and non-primate lentiviruses. Further work will be needed to understand the requirement of these charged rings for divergent lentiviruses.

## Variability in HIV-1 utilization of capsid-dependent host proteins

The HIV-1 capsid directly interacts with many host proteins to promote post-entry processes. Genetic, biochemical, and structural studies have discovered two major protein interfaces on the HIV-1 capsid. One interface involves CA binding to cyclophilin A (CypA), while the other is important for capsid interactions with multiple host proteins represented by CPSF6 and NUP153 (Fig. 1). Recent work on these capsid-binding host proteins have uncovered how sequence variation in these interfaces affects viral infectivity as well as other phenotypic properties of HIV-1.

### CypA and NUP358

CypA binds to an exposed proline-rich loop between helixes 4 and 5 (H4/5L) in the CA-NTD [104]. The H4/5L can also interact with the cyclophilin domain of NUP358, a component of the nuclear pore complex (NPC) [105, 106]. Most mutations in the H4/5L do not affect virus particle production and mature capsid formation [107], although some were shown to affect core maturation [55, 108]. Phenotypic changes caused by amino acid substitutions in the H4/5L mostly manifest during post-entry processes.

One group of amino acid changes in the H4/5L block CA-CypA binding. Key amino acids that participate in CA binding to CypA are G89 and P90 residues in the H4/5L [109]. This dipeptide motif is highly conserved among HIV-1 strains. Amino acid changes at these positions (e.g., G89A/V, and P90A) as well as those adjacent to this motif can reduce CA-CypA binding and the amount of CypA incorporated in virus particles [110–113]. These CypA-binding mutations inhibit viral infection and propagation in multiple cell types [111, 112, 114–118].

These mutant viruses deficient for CypA binding are blocked at reverse transcription [21, 111, 118–120]. The P90A mutation did not overtly alter the amount of pelletable CA in a fate-of-capsid assay [107]. In contrast, CypA-binding mutants displayed distinct intracellular trafficking patterns; G89V particles traffic in the cytoplasm at a higher rate of speed than WT particles [121], whereas the P90A mutant facilitates faster docking of intracellular particles at the NPC [92], suggesting that CypA slows down PIC nuclear entry. The capsid is a viral determinant for integration targeting [122]. Both G89V and P90A mutations increase the frequency of integration within transcription units [105], but there have been few studies investigating how the altered integration sites by disrupting CA-CypA interactions affect post-integration processes.

HIV-1 exploits cellular proteins to promote its nuclear entry [6, 123]. A major consequence of disruption of CA-CypA interactions is the change in HIV-1 utilization of nuclear entry cofactors [94, 105]. NUP358 is a RAN-binding cellular protein localized to the cytoplasmic side of NPCs and processes a domain that is homologous to CypA and capable of interacting with incoming capsids [105, 106]. G89V and P90A substitutions render HIV-1 less sensitive to NUP358 depletion [94, 105, 124]. Additionally, HIV-1 infection causes localization of NUP358 into the cytoplasm, but this phenotype is abolished when incoming virus harbor the P90A change [125]. While these reports provided support for the role of the cyclophilin domain of NUP358 for HIV-1 nuclear entry, one report showed that it can be dispensable in certain contexts [126]. NUP153 is another major host protein involved in HIV-1 nuclear entry. CypA-binding mutants became insensitive to NUP153 depletion [94, 95, 124]. A recent report showed that transportin-1 (TRN-1), a transporting receptor, is required for HIV-1 infection. Interestingly, the G89V mutation, but not the P90A mutation, decreased TRN-1 dependence of HIV-1 infection [127]. TRN-1 alters the tube structure generated by recombinant CA-NC proteins for the WT virus but not the G89V mutant, suggesting direct CA binding to TRN-1 mediated by the G89 residue [127].

CypA-binding mutations also modulate the sensitivity of HIV-1 to host antiviral proteins. Two well-studied groups of such restriction factors are TRIM5α and MX2. In general, previous studies demonstrated that CypA-binding mutant viruses are less sensitive to these antiviral proteins (discussed below). There are other host proteins that can block HIV-1 in a manner dependent on the H4/5L. SUN1 is a nuclear membrane protein of which ectopic expression blocks infection of the WT virus, but not the G89V mutant [128]. Additionally, an HIV-2 variant to enhance CA binding to CypA is blocked by a mechanism involving SUN2 [129]. Daxx is another example. The Daxx protein can block HIV-1 infection at reverse transcription [130] but displays differences in antiviral activity between WT and P90A viruses [131]. Proteomic work showed that HIV-1 cores are part of the multi-protein complex composed of Daxx, TNPO3, TNPO1, TRIM5α and TRIM34, but the P90A substitution decreased the level of capsid associations with these host molecules [131].

Another phenotype altered by these CypA binding mutations is innate sensing of HIV-1. Previous reports suggested that the HIV-1 capsid protects newly synthesized DNA from recognition by host DNA sensors, such as cGAS [132, 133]. Experimental evidence suggested a link that connects CypA–CA interactions with cGAS-dependent sensing of HIV-1 DNA. Namely, both G89V and P90A mutants were shown to induce an elevated level of type I interferons (IFNs) in macrophages [133–136]. Similarly, an HIV-2 variant engineered to enhance its CypA binding ability activates innate immune sensing by cGAS [132]. It was also reported that HIV-1 infection of dendritic cells using Vpx to relieve SamHD1 restriction resulted in the maturation of dendritic cells but the G89V mutation eliminated this immunostimulatory ability [137].

Amino acid changes in the H4/5L also modulate the sensitivity to antiviral activity mediated by type I IFN activation in target cells. The P90A mutant was more sensitive to IFN-α-induced blocks in THP-1 cells [138]. Additionally, an HIV-1 variant carrying multiple substitutions in the H4/5L displayed enhanced IFN-β sensitivity in T cells [139]. Both viruses are deficient for CypA binding, but the lack of CypA binding may not be the mechanism of the increased sensitivity to IFN-mediated inhibition, as HIV-1 was able to acquire resistant to IFN-β-mediated restriction without restoring the binding ability to CypA [139].

Another group of amino acid changes in the H4/5L, which are represented by A92E and G94D, cause a phenotype distinct from those by CypA binding mutations. A92E and G94D mutations emerged after HIV-1 replication in the presence of an analog of CsA, which block CA–CypA interactions [140]. These mutant viruses became not only resistant to CsA but also dependent on CsA for efficient replication. Another key characteristic of these CsA-dependent mutants is their reduced ability to infect non-dividing cells [141–143]. The CsA-resistant/dependent phenotype can be conferred by amino acid changes outside of the H4/5L (e.g., T54A) [141, 142, 144, 145], and is also observed in primary isolates [146–149].

The CsA dependence of these mutants appears to be caused by an inhibitory effect of CypA-CA interactions, since other means to disrupt these interactions improve their infectivity [107, 114, 143, 150–153]. However, how these CsA-dependent mutations are blocked by CA–CypA interactions is somewhat uncertain. Investigation of these CsA-dependent mutants with respect to their reverse transcription, nuclear entry, and capsid disassembly has generated conflicting results between different studies [81, 107, 141–143, 153]. One consistent observation is that infectivity of these mutants is rescued upon CsA treatment several hours after infection [141–143], suggesting that intracellular subviral particles are not immediately destroyed by CypA-mediated restriction.

These mutants are blocked only in certain cell types [107, 114, 143, 150–153]. A model to explain this cell-type-dependence is that CsA-dependent mutants are restricted by a host cellular factor [143, 152, 153]. A92E and G94D mutants are not hypersensitive to human TRIM5α [154, 155] and display reduced sensitivity to MX2 [156, 157]. CsA-dependent mutants, including G94D and T54A, can be rescued by mutations (N74D and A105T) that prevent CA binding to CPSF6 [144, 149, 158, 159], suggesting that endogenous CPSF6 can act as an antiviral molecule against CsA-dependent mutants. Infectivity of CsA-dependent mutants is restored by RNAi-mediated depletion of CPSF6 in one study [149] but not in another study [124]. Additionally, there was no difference in CPSF6 levels between permissive and non-permissive cells [160].

The H4/5L is polymorphic among clinical HIV-1 variants, harboring naturally occurring substitutions, such as H87Q, I91V, and M96I. The H87Q substitution often arises among HIV-1 variants resistant to antiretroviral therapy during the late stage of viral infection [161] or in CTL escape mutants from patients carrying HLA B57 alleles [162]. Viruses carrying these amino acid changes replicate at levels comparable to representative lab-adapted strains. Interestingly, in a head-to-head competition, H87Q became dominant after viral replication in peripheral blood leukocytes [163]. Some, but not all, changes reduce CA binding to CypA; the H87Q mutation reduces CA–CypA binding by fivefold, whereas the I91V mutation causes a negligible change [113]. Importantly, these amino acid changes can relieve CypA dependence of HIV-1 replication [147, 164–167]. The CypA independence of HIV-1 infection was observed in 15% of HIV-1 clinical strains [166].

How these naturally occurring polymorphisms in the H4/5L affect biological properties of HIV-1 has been begun to be investigated. Three amino acid changes (H87Q, A88P, and I91V) were shown to increase capsid stability in the fate-of-capsid assay [135]. Alterations in capsid stability can result in differential innate immune responses in myeloid cells [90, 168, 169]; however, these naturally occurring changes did not lead to an elevated level of IFN induction in macrophages [135]. The primary strain 93BR020 differs from the prototypic HIV-1 strain in four amino acid residues (V86T/H87Q/A92P/M96I) but is identical to the WT virus in their ability to bind to CPSF6 and utilize NUP358 [170].

Comparative studies of phylogenetically diverse groups of lentiviruses have revealed a complex interplay between CypA and CA during lentiviral evolution. Differences in HIV-1 utilization of CypA are illustrated by HIV-1 Group O, a group of variants phylogenetically distinct from major HIV-1 variants (called Group M) [171, 172]. Some Group O isolates are resistant to drugs that block CA–CypA interactions [166, 167, 173]. Furthermore, the CypA-binding loop of Group O confers the CsA-resistance/dependent phenotype [150]. The CypA-binding loop of a Group O isolate reduces CypA binding affinity [113]. Another study showed that a region outside of the H4/5L contributes to the distinct CypA requirement by HIV-1 group O isolates [172].

HIV-2 is distinct from both groups of HIV-1 in its dependence on CypA for infection. HIV-2 does not incorporate CypA or incorporate it at a reduced level in virions when compared to HIV-1 [110, 171]. Similar to HIV-1 group O variants, HIV-2 is insensitive to CypA inhibitors and replicates efficiently in cells deleted of CypA [115, 173–175]. A genetic determinant is the CypA-binding loop, since its transfer to HIV-1 reduces CA interactions to CypA and also relieves the dependence of HIV-1 infection on CypA [173]. However, a subset of primary HIV-2 variants display an infectivity defect in CypA-deficient cells [176], suggesting that CypA utilization varies between HIV-2 variants.

CypA utilization is not a conserved property of lentiviruses but also not a trait specific for HIV-1. Early studies showed that the Gag protein from certain SIV strains do not bind to CypA [104]. Consistently, viruses that belong to the SIVsmm/mac lineage as well as two strains of SIV from African green monkeys do not package CypA in virus particles [110, 171, 177, 178]. These SIV strains replicate in a CypA-independent manner [115, 166, 171]. On the other hand, genetic and biochemical evidence showed the ability of capsids from a variety of lentiviruses to physically interact with CypA, including FIV, SIVcpz, SIVagmTan, SIVgsn, and SIVmnd1 [155, 179–183]. However, their CypA dependence for infection may differ from HIV-1, as some of these viruses appear to be insensitive to inhibitory effects of CsA or related compounds [166, 184]. The difference between HIV-1 and other CypA-binding viruses is also observed for NUP358 utilization; FIV binds to both CypA and the CypA domain of NUP358, but is resistant to NUP358 depletion [126, 185]. Additionally, the cyclophilin domain of NUP358 differs in protein sequence between primates. Such differences also lead to differential interactions between NUP358 and different lentiviral capsids, which were proposed to be a mechanism to drive HIV/SIV evolution during cross-species transmission [186].

### CPSF6 and NUPs

CPSF6 is another capsid-binding protein that regulates post-entry processes [93]. CPSF6 binds to a preformed pocket generated by the NTD-CTD interface with a higher affinity to a multimerized form of CA than CA monomers [187–189]. Interestingly, HIV-1 uses this binding pocket to interact with other proteins, such as NUP153 and Sec24C [95, 188–190]. Amino acid substitutions in this CPSF6-binding pocket alter a wide variety of phenotypic properties of HIV-1 and are instrumental in understanding how HIV-1 subviral complexes traverse an intracellular environment to complete the infection process by inserting proviral DNA into the host chromatin.

A broad range of phenotypic outcomes caused by mutations in the CPSF6 binding pocket is illustrated by an N74D substitution, which was originally identified after HIV-1 passage in cells expressing a truncated form of CPSF6 (CPSF6-358) [93]. The side chain of N74 forms a hydrogen bond with CPSF6 [187–189]. The N74D mutation confers resistance to antiviral activity of CPSF6-358 [93, 187, 191] and blocks capsid binding to CPSF6 [93, 187, 192]. Imaging work showed colocalization of HIV-1 with CPSF6 but this was prevented by the N74D mutation [121, 193, 194].

The N74D mutant has garnered a great deal of attention, since its infection is independent from HIV-1 nuclear entry co-factors utilized by the WT virus. Namely, infection of the N74D mutant is insensitive to depletion of NUP358 and NUP153 [93, 94, 105]. Additionally, the N74D change alters virus utilization of a comprehensive panel of nucleoporins and nuclear entry cofactors [124, 195, 196]. NUP153 binds to the capsid at the same interface as CPSF6 [95, 188, 189]. However, NUP153 binding is not correlated with NUP153 dependence for infection, as the N74D mutant, which is independent from NUP153, displays hyper-binding activity to NUP153 [95, 197]. The N74D virus maintains the ability to infect non-dividing cells [93, 95, 124, 197]. This observation suggests that PICs need to translocate through nuclear pores, and points to a flexibility in how HIV-1 subviral complexes gain access inside the nucleus [93].

Mutant virus bearing the N74D substitution infects immortalized cells without any overt defect [93, 187]. In contrast, the N74D mutant is impaired in primary CD4 + T cells and macrophages with decreased levels of revere transcription products and 2-LTR circle formation [133, 159, 198–201]. Interestingly, replication of HIV-1 containing the N74D mutation in macrophages is partially restored by treatment of antibody to the IFNα/β receptor α chain as well as capsid-binding small-molecule inhibitors PF74 and BI-2 [133, 198]. HIV-1 infection of macrophages with the N74D mutant was reported to stimulate type I IFN induction [133, 135]. In other studies, the N74D virus did not enhance innate immune responses when used to infect macrophages and CD4 + T cells [199, 202].

The intracellular behavior of the N74D virus has been extensively studied using various experimental approaches. While minor differences exist between studies, a number of distinct phenotypic changes caused by the N74D mutation have been consistently observed across multiple studies. The N74D mutant differs from the WT virus in intracellular trafficking and capsid disassembly. For instance, N74D particles display faster cytoplasm trafficking than WT particles in macrophages [121] and uncoat at the nuclear envelope [83, 194]. The exact timing of HIV-1 uncoating is still a matter of debate [71, 73, 83, 194, 203–208]. However, the N74D mutant, when compared to the WT virus, appears to delay capsid uncoating in various assays [73, 81, 83, 84]. Imaging and biochemical studies showed that fewer numbers of PICs or CA reside in the nucleus for the N74D mutant than the WT virus [206, 209, 210]. The N74D mutant virus also differs from the WT virus in nuclear localization. Subviral complexes of viruses carrying a mutation of N74 (N74A or N74D) reside near the nuclear periphery or lamina [83, 209]. Consistent with these findings, transcription sites of the N74D virus are closer to the nuclear envelope than those of the WT virus [194].

CPSF6 plays a key role in directing HIV-1 integration sites toward active chromatin [211], including genes linked to speckle associated domains [212, 213]. This unique integration targeting is abolished by the N74D mutation [105, 197, 199, 211, 214]. How such integration site change alters the level or dynamics of HIV-1 transcription remains unclear. Sadowski et al. used dual reporter virus to find that the N74D virus is the same as the WT virus with respect to the proportion of productive cells, which are active in LTR-driven viral gene expression [215].

The N74D change modulates capsid interactions with other host factors as well. Recent work discovered that Sec24c is a novel capsid interacting protein that promotes early HIV-1 infection [190]. Remarkably, Sec24c binds CA at the binding interface utilized by CPSF6 and NUP153 [190]. The N74D mutation impedes CA binding to the FG motif of Sec24C and is insensitive to Sec24c depletion [190]. The N74D mutation prevents cytoplasmic localization of NUP358 and relieves the requirement of KIF5B for HIV-1 infection [125]. Capsid interactions with CPSF6 also regulate CypA utilization by HIV-1 during post-entry processes, as the N74D mutation was shown to increase the dependence of HIV-1 infection on CypA [159].

Another well studied mutation in the CPSF6 binding pocket is an A77V substitution. The A77V mutation is the HIV-1 counterpart of an A76V replacement in SIVmac CA, which arose after adaption of a chimeric HIV-1 carrying SIVmac CA [216]. The A76V mutation in SIVmac and the A77V mutation in HIV-1 confer resistance to inhibition by CPSF6-358 [199, 360]. The A77V mutation reduces CA binding to CPSF6 [199]. The A77V virus shares many similarities with the N74D virus. Some common properties between these CPSF6-binding mutants include their altered dependence on nuclear entry factors, uncoating at the nuclear envelope and changes in integration targeting [194, 199, 201, 212, 213, 217].

A key difference from the prototypic N74D mutation is that the A77V mutant virus retains a nearly WT-level of infectivity in primary cells [199, 201, 218]. This difference between these two mutants may be explained by differential sensitivity to TRIM34, which blocks the N74D virus, but not the A77V virus [219]. The A77V virus replicated in humanized mouse as efficiently as the WT virus [199]. However, the A77V CA reverted back to the WT CA in multiple animals [199]. These observations suggest that optimal CPSF6 binding to the viral capsid is not an absolute requirement for HIV-1 replication but confers a moderate but significant fitness advantage to CPSF6-binding viruses.

The A77V mutation has been used a tool for cell-based imaging studies of CPSF6 functions in primary cells, which substantiated earlier observations made with the N74D mutation in immortalized cells. For instance, A77V subviral complexes accumulate at the nuclear envelope and are associated with NPCs [201]. Additionally, A77V infection resulted in a reduced number of HIV-1 subviral complexes in the nucleus detected by CA and viral DNA signals [201]. Zila et al. leveraged the ability of the A77V mutation to increase the steady-state accumulation of virus complexes at nuclear pores to visualize cone-shaped capsids inside the NPC central channel and their morphological alterations [208].

The N57 residue in the helix 3 is a key amino acid residue involved in capsid binding to CPSF6 and other host factors [95, 187, 188], and its mutations change several phenotypic characteristics differently from both N74D and A77V mutations. The N57A substitution abrogates CA binding to CPSF6 and renders HIV-1 insensitive to CPSF6-358 [95, 187, 191, 197]. Similar to other CPSF6 binding mutations, the N57A mutation also blocks CA binding to Sec24c [190]. The N57A mutation alters nuclear entry and integration targeting [105, 197]. The N57A mutant also resembles the N74D mutant in that their infectivity defect is exacerbated in the presence of CsA [105], suggesting a link between CypA and CPSF6 binding to CA. In vitro evolution of virus harboring the N57A substitution resulted in selection for a compensatory mutation of G94D in the CypA-binding loop [220]. Importantly, the G94D variant by itself is also rescued by the N74D substitution [159], underscoring similarities between N57A and N74D mutations.

A key difference between positions 57 and 74 is their mutational effects on CA binding to nucleoporins. N57 mutations (N57A/D/S) block CA interactions with NUP153 [95, 197] as well as other nucleoporins including NUP62, NUP98, and NUP214 [197]. Another fundamental difference is that several amino acid changes at position 57 impair the ability of HIV-1 to infect non-dividing cells [24, 89, 95, 105, 197]. It is possible that N57A’s loss of NUP binding may account for the relative lack of these mutants to infect cells independent from cell cycle progression [95, 197]. Finally, virus carrying the N57A change is severely defective in primary CD4 + T cells and macrophages [89, 220], a property shared by N74D but not A77V mutations. However, unlike the N74D virus, the N57A virus is not restricted by TRIM34 [219].

Other mutations in the CPSF6 binding pocket (M66F, Q67A, K70A, A105S/T, T107A/I, and K182R/A) also affect capsid binding to CPSF6 [138, 149, 187, 188, 191, 199], while amino acid changes outside of this CPSF6 binding pocket were shown to change the sensitivity of HIV-1 to restriction by CPSF6-358 [139, 221]. CPSF6 binding generally correlates with HIV-1 dependence on nuclear entry co-factors, such as NUP358 and NUP153. As such, most of the variants harboring amino acid changes in the CPSF6 binding pocket reduce the sensitivity to depletion of NUP358 and TNPO3 [149, 191, 222]. The A105T mutant was also shown to share a similar nuclear phenotype with the N74D and A77V mutants in that their integration occur at the nuclear periphery [209].

It is notable that amino acid replacements at some of these positions, in particular positions 105 and 107, repeatedly emerged after virus propagation in different contexts. For instance, the A105T change was identified after serial passage of the T54A mutant virus, a CsA-resistant/dependent virus  [144]. The T107I mutation was identified after in vitro evolution of a CTL escape variant that lost the cell-cycle independence for infection [149]. As we will describe below, other studies identified drug resistant mutations at these positions [223–225]. Thus, these two amino acid residues (A105 and T107) are among those in CA that are most tolerable to mutational changes.

Amino acid residues in the CPSF6 binding pocket are not completely conserved among diverse strains of lentiviruses. Previous work demonstrated that different lentiviruses vary in their utilization of host factors that bind to the CPSF6 binding pocket. HIV-2 and closely related SIVmac are a group of viruses that exploit CPSF6 for viral propagation. CA of HIV-2 and SIVmac was shown to bind to CPSF6 [187]. Key mutations in the CPSF6 binding pocket, such as N56A and N73D (HIV-2/SIVmac numbering), indeed conferred resistant to CPSF6-358 [187]. In contrast, HIV-2 and SIVmac appear to be less dependent on the K182 residue for CPSF6 binding than HIV-1 [226].

In contrast to primate lentiviruses, non-primate lentiviruses, such as bovine immunodeficiency virus (BIV), FIV and EIAV, are resistant to cytoplasmic CPSF6 [93, 227]. Consistently, non-primate lentiviruses did not show a strong preference for speckle associated domains as integration sites [213]. Furthermore, CPSF6 absence did not strongly affect integration targeting of non-primate lentiviruses [213]. Sec24c is nearly identical to CPSF6 in its requirement for infection by diverse lentiviruses, as EIAV and FIV were not affected by Sec24c knockout in contrast to some SIV strains of which infectivity were reduced in cells lacking Sec24c [190].

NUP153 utilization by related lentiviruses exhibits a pattern slightly different from that for CPSF6. SIVmac, SIVagmSab and SIVagmTan are sensitive to inhibition by NUP153 knockdown [95]. However, NUP153 requirement for infection is not conserved among primate lentiviruses, as the sensitivity of NUP153 depletion drastically vary among circulating SIV strains [184]. Non-primate lentiviruses, FIV and BIV, are resistant to NUP153 knockdown without any binding activity. In contrast, EIAV has an appreciable ability to interact with NUP153 and utilizes it for infection [95]. Interestingly, EIAV CA binds to NUP153 in a manner different from HIV-1 [95]. Overall, EIAV and FIV were shown to be less sensitive to inhibitory effect of depletions of multiple nuclear entry factors [93, 124]

## Capsid-targeting antiviral molecules

A variety of molecules directly bind to the HIV-1 capsid and block viral infection. These capsid-targeting antiviral molecules can be either host-encoded restriction proteins or small-molecule compounds. Two major groups of capsid-dependent host factors that will be discussed below are TRIM5 and MX2. On the other hand, previous drug development efforts culminated in the discovery of several small-molecule compounds that block capsid-related functions with different mechanisms of action. Extensive research in the past has elucidated how these antiviral molecules recognize the viral capsid and interfere with capsid-mediated processes. Furthermore, comprehensive characterization of escape variants that acquired resistance to these capsid-dependent antiviral molecules has illuminated the viral evasion mechanism and identified sequence elements of CA that are vulnerable to mutational effects and those that possess adaptive potential.

### TRIM5

TRIM5 proteins are capsid-dependent restriction factors and consist of TRIM5α and TRIMCyp [228–233]. TRIM5 directly binds to the HIV-1 capsid and accelerates uncoating to prevent reverse transcription [75]. TRIM5α, which has evolved under positive selection in primates [234], is a major determinant for species-specificity of retroviruses [235–237]. For instance, human TRIM5α, while ineffective for HIV-1, potently blocks infection by N-tropic murine leukemia virus (N-MLV) or EIAV. In contrast, HIV-1 infection is severely impaired by TRIM5α from Old World monkeys. Past studies used both in vitro and in vivo model systems to reveal how sequence variation in CA among HIV-1 and other lentiviruses influences the sensitivity to restriction mediated by TRIM5 proteins.

HIV-1 is blocked by TRIM5α from many species of Old World monkeys. Previous work frequently relied on restrictive alleles of TRIM5α from rhesus macaques (RMs) in order to map the viral determinant of HIV-1 restriction by TRIM5α. These studies demonstrated that HIV-1 restriction by TRIM5α is mediated by multiple interfaces exposed on the external surface of the viral capsid [237, 238].

A flexible loop between α-helices 4 and 5 of CA (H4/5L), which binds to CypA, is one major determinant of TRIM5α restriction of HIV-1 [235, 239]. Replacement of the HIV-1 H4/5L with the corresponding sequence from SIVmac, an RM-derived SIV strain insensitive to restriction by RM TRIM5α, restored HIV-1 infectivity in monkey cells [240, 241]. Single amino acid changes in the H4/5L also enhance HIV-1 infection in cells expressing RM TRIM5α [240, 242, 243]. One such change, H87Q, is present in circulating HIV-1 strains [161]. Similarly, CA from a certain type of naturally occurring HIV-1 variants enables HIV-1 to evade restriction in simian cells [164, 244]. The role of the H4/5L in capsid recognition by TRIM5α was also supported by in vitro HIV-1 evolution under selective pressure from RM TRIM5α [243, 245, 246]. HIV-1 variants that had adapted to restriction by RM TRIM5α gained amino acid replacements within the H4/5L (e.g., V86M, H87Q, V86E, I91N, and I91T). The V86M mutation was shown to reduce capsid binding to RM TRIM5α while maintaining binding with CypA [245]. A TRIM5α-resistant mutation was also found outside of the CypA-binding motif but located within the H4/5 loop (R100S in HIV-1 numbering) [247].

A second region of the capsid surface that confers resistance to TRIM5α involves helix 6. This region contains an exposed loop formed by the C-terminal portion of helix 6 and a mini-β-hairpin that connects helices 6 and 7 [240]. This region also includes a ridge formed at the interface of helices 3 and 6 (helix 3/6 ridge) [242]. Amino acid changes at position 116 enhance HIV-1 infection in RM TRIM5α-expressing cells [240, 243]. One such mutant, G116E, was indeed shown to resist TRIM5α-mediated disruption of HIV-1 cores [243]. Notably, the CA 116th amino acid residue is polymorphic among HIV-1 strains, suggesting that it plays a role during viral adaptation. Interestingly, this position is one of the determinants for MX2 restriction of HIV-1 (discussed below). A Q112D substitution in helix 6 is another mutation that was shown to improve replication of monkey-tropic HIV-1 in simian cells [247, 248]. Additionally, viruses containing various mutations in the helix 3/6 ridge were shown to be more infectious in simian cells [242].

The N-terminus of CA has a β-hairpin structure, which is surface exposed and modulates HIV-1 sensitivity to TRIM5α. Amino acid replacements, such as N5D, Q7A/Q9A, M10I/L/V, M10L, and M10V, conferred partial resistance to RM TRIM5α [240, 243]. A similar observation was made in work studying the sensitivity of HIV-2 or SIVmac239 to monkey TRIM5α [249]. Additional support for the role of this β-hairpin structure as a recognition motif for TRIM5α came from work examining the consequence of adaptation of N-MLV to RM TRIM5α [250]. This work identified several TRIM5α resistant mutations, which include a L10W change in the β-hairpin loop. Additionally, gain-of-sensitivity experiments in which a resistant SIVmac strain was engineered to acquire the sensitivity to restriction by RM TRIM5α suggested that the CPSF6 binding pocket can also influence HIV-1 restriction by TRIM5α [251].

Overall, these findings suggest that TRIM5α recognizes multiple epitopes exposed on a large surface of the viral capsid [243, 247, 248, 250–253]. This idea based on mutational studies has been also supported by biochemical and structural studies [254–261]. Notably, these resistant mutations, when individually introduced to HIV-1 CA, only confer partial resistance to RM TRIM5α. Several studies demonstrated that a combination of amino acid changes on the exposed surface are required for rendering HIV-1 fully resistant to RM TRIM5α. Considerable efforts have been made to generate an HIV-1 variant that completely evades restriction by RM TRIM5α while retaining the full replicative capacity, as such virus would serve as an invaluable tool for improving the macaque model of HIV-1 [262, 263]. Several laboratories harnessed the knowledge obtained from these studies and successfully achieved the goal to engineer the viral capsid that enables HIV-1 variants to efficiently replicate in RM cells [243, 247, 264].

TRIMCyp is a chimeric protein between TRIM5α and CypA and encoded by certain species of Old World and New World monkeys [232, 233, 265–269]. The CypA domain of TRIMCyp mediates capsid recognition by interacting with the H4/5L of CA, which contains the CypA-binding motif. As expected from this binding mechanism, individual or a combination of mutations in the H4/5L (e.g., H87Q, and G89V) are able to enable HIV-1 to infect TRIMCyp-expressing cells [164, 165, 232, 233, 270].

Antiviral activity of TRIMCyp varies among different species. Owl monkey TRIMCyp differs from its counterparts from Old World monkeys. For instance, previous studies showed that TRIMCyp in Old World monkeys originated through an independent event of retrotransposition of the CypA sequence in the 3′untranslated region of the TRIM5 gene [265–269]. Additionally, TRIMCyp from cynomolgus macaque (CM) is polymorphic and this polymorphism affects susceptibility to HIV-1 infection [271–273]. Namely, HIV-1 infection was blocked by CM TRIMCyp carrying aspartic acid (D) and lysine (K) at positions 369 and 446 (denoted as the DK haplotype) [271–273], but not by another haplotype of CM TRIMCyp carrying asparagine (N) and glutamic acid (E) in corresponding residues (denoted as the NE haplotype) [271–273]. In contrast, HIV-2 can be effectively blocked by TRIMCyp (NE). The differential sensitivity to restriction by TRIMCyp (NE) is governed by the 88th residue of CA [274].

As discussed above, HIV-1 can fully evade TRIMCyp restriction by just a single amino acid substitution. However, an HIV-1 variant that acquired resistance to TRIMCyp presented a more complicated scenario [275]. In this study, a combined approach of random mutagenesis and in vitro adaptation was used to yield an HIV-1 clone with high resistance to CM TRIMCyp [275]. The adapted variant was shown to carry four amino acid changes in the H4/5L (H87R, A88G, P90D, and P93A) in addition to a Q112D change in helix 6 of CA-NTD. Notably, this CA variant is completely resistant to TRIMCyp-mediated restriction, whereas the P90A CA mutant remains partially sensitive to TRIMCyp [139]. Thus, in two studies using similar combinatory approaches to generate HIV-1 variants resistant to RM TRIM5α or CM TRIMCyp [243, 275], multiple amino acid replacements in HIV-1 CA were required to overcome antiviral activity by these TRIM5 proteins. An additional study showed that this TRIMCyp-resistant variant gained unusually high sensitivity to type I IFNs [139]. It is currently unknown whether the increased sensitivity to type I IFNs is a common property of HIV-1 variants that acquired the resistance to simian TRIM5 proteins.

Studies of cross-species transmissions of African SIV strains to Asian macaques have also revealed how the SIV capsid adapts to the selection pressure imposed by TRIM5 proteins [276]. A well-used model is RM infection with primary or minimally passaged SIV strains from sooty mangabeys (SIVsmm), which are sensitive to restriction by RM TRIM5α and TRIMCyp. In vivo experiments uncovered a central role of TRIM5 proteins in the susceptibility to SIVsmm infection and the evolution of SIV capsid [277, 278]. Specifically, these studies identified CA mutations that allow escape from viral suppression by TRIM5 proteins [277, 278]. Interestingly, the viral adaptation in this model appears to be achieved through a common evolutionary trajectory; two amino acid substitutions at positions 37 (P37S) and 98 (R98S) were found in multiple animals infected with SIVsmm [277, 278]. Introduction of these two changes together increased SIVsmm infectivity in cells expressing a restrictive haplotype of RM TRIM5α in vitro [278]. These adapted changes were further validated by in vivo studies in which an SIVsmm variant carrying both P37S and R98S mutations caused higher viremia and lower survival rate than the parental strain in infected animals [279]. Note that the R98S mutation located at the bottom of the H4/5L serves as a primary residue to increase HIV-1 resistance to RM TRIM5α [247]. In contrast, it is unclear how P37S confers resistance to RM TRIM5α. A possible mechanism is that this mutation may alter capsid stability, since an amino acid change of the corresponding position in HIV-1 reduces capsid stability [61].

Similar studies using the macaque model have yielded new insights into SIVsmm adaption to TRIMCyp. In these studies, the authors found that SIVsmm overcame TRIMCyp restriction by acquiring amino acid changes in the H4/5L (e.g., Q85P, G87Q, L90P, and A92P) [280]. As expected, these mutations rendered the parental SIVsmm strain resistance to RM TRIMCyp. Remarkably, SIVsmm found a way to evade restriction imposed by both TRIM5α and TRIMCyp; SIVsmm passaged in an animal expressing both TRIM5α and TRIMCyp accumulated mutations in the H4/5L as well as the aforementioned P37S and R98S changes. [278]. Many instances of successful adaptation of TRIM5-sensitive SIVsmm to RMs are in stark contrast to the difficulties in enforcing HIV-1 to overcome the species barrier to Asian macaques.

Human TRIM5α was generally considered to be a weak inhibitor of HIV-1 infection [238]. However, some circulating HIV-1 variants, especially those isolated from protective alleles of HLA, exhibit an elevated level of sensitivity to restriction by human TRIM5α [148, 281–283]. HIV-2 is also more sensitive to human TRIM5α than HIV-1 [176, 284, 285]. Interestingly, a large-cohort analysis in Guinea-Bissau suggested that naturally occurring polymorphisms at position 119 or 120 in CA can influence the viral load in HIV-2-infected individuals [286]. Specifically, CA sequences of HIV-2 from individuals with low viral loads had a proline at position 119 or 120, whereas those from individuals with high viral loads tended to have non-proline residues [286]. Critically, in vitro studies showed that these positions in HIV-2 CA affect the sensitivity to human and CM TRIM5α [287, 288], although one study did not find a similar mutational effect when a different CA sequence was used [285].

Recent reports have revealed that human TRIM5α significantly contributes to IFN-mediated antiviral effects on HIV-1 infection and that CypA protects HIV-1 from TRIM5α restriction in primary blood cells [118, 120, 289, 290]. Consistent with these findings, infectivity of CypA-binding deficient mutants, G89V and P90A, was impaired in primary cells but restored by TRIM5α depletion [118, 120]. These findings argue that human TRIM5α is a critical component of the host antiviral system that affects the evolution of the HIV-1 capsid. Consistent with this idea, previous work demonstrated that amino acid replacement with certain low-frequency residues at sites that are under positive selection increased the sensitivity of HIV-1 to human TRIM5α, suggesting that the majority of CA sequences of circulating HIV-1 variants belonging to clade B had optimally adapted to human TRIM5α [291]. New experimental protocols used in these recent studies [118, 120] will be a valuable tool to further study how sequence variation in the H4/5L and other exposed sequence elements dictates differential sensitivity to human TRIM5α among different variants of the HIV-1 and HIV-2 lineages.

### MX2

MX2, a member of the dynamin-like GTPase family, is a capsid-dependent HIV-1 restriction factor that is induced upon activation of type I IFN signaling [156, 292, 293]. MX2 is localized to the nuclear envelope and blocks HIV-1 nuclear import [156, 157, 292, 293]. Among several distinct domains of the MX protein, the N-terminal tail is important for localization to the NPC and for antiviral activity [124, 294–298]. MX2 can directly bind to the viral capsid [134, 299–302]. Importantly, the MX2 binding interface of HIV-1 is not present in individual building blocks of HIV-1 capsid, such as CA hexamers, but composed of a structure that is only present in the high-order CA lattice [261, 299–301, 303]. Many amino acid changes that confer MX2 resistance on HIV-1 have been discovered among well-utilized CA mutants, circulating HIV-1 variants and passaged viruses that had been adapted in MX2-expressing cells [156, 157, 292–294, 300]. These MX2-resistance mutations are scattered over the entire sequence of CA, suggesting multiple evasion mechanisms of HIV-1.

A major determinant of MX2 resistance involves the CypA-binding loop. Well-characterized G89V and P90A mutations as well as other amino acid changes (H87R, P90T, and Q95L) in a CA mutant library confer MX2 resistance [134, 156, 157, 292–294, 300, 304]. Virus evolution experiments identified an A88T substitution in the CypA-binding loop, which allows HIV-1 to escape from MX2 restriction [293]. MX2 sensitivity differs among circulating HIV-1 variants and the difference is often determined by naturally occurring polymorphisms in the CypA-binding loop (e.g., V86A/Q, H87P/Q, A88V and A92P) [134, 304]. Many MX2-resistant mutations in the CypA-binding loop diminish CA interactions with CypA. Indeed, MX2 restriction of HIV-1 can be relieved by CsA or CypA depletion [124, 170, 293]. However, CypA-binding mutations did not significantly reduce CA binding to MX2 [299, 300, 303, 304]. Additionally, there is evidence suggesting that CypA recruitment by itself is not essential for the full sensitivity to MX2 restriction [139, 170]. An adapted HIV-1 variant completely lost CypA binding but remained sensitive to MX2 [139]. Likewise, recent work showed that addition of the A92E mutation to the P90A mutant partially rescues MX2 sensitivity [170]. As the A92E mutation can reduce the dynamics of the CypA-binding loop [305], a role of the stabilization of the CypA-binding loop was suggested as a mechanism of MX2 restriction of HIV-1 [170].

The CypA binding loop is exposed on the surface of HIV-1 cores. Two other known exposed loops also affect MX2 restriction of HIV-1. Specifically, a mutation in the N-terminal β-hairpin (M10I mutant) confers resistance to MX2 whereas a Q4R mutation can sensitize MX2 restriction [139, 294]. The G116 residue in the helix 6 is another residue of which amino acid changes (G116A/Q/R) diminish MX2 sensitivity of naturally occurring variants [134, 170, 283]. Characterization of a primary strain that is resistant to antiviral activity of MX2 (93BR020) showed that the amino acid at position 116 acts together with a histidine at position 120 and the CypA binding loop to influence MX2 sensitivity of HIV-1 [170].

Another group of MX2 escape mutations are located within the CPSF6 binding pocket, including N57S, K70R, N74D and A105T mutations [134, 156, 157, 292, 300]. A T107I substitution occurred in animal-to-animal passage experiments of a simian-tropic HIV-1 variant and was shown to be linked with MX2 resistance [306]. The T107I mutation reduces CA binding to CPSF6 and impairs virus replication [306]. How CPSF6 binding deficient mutants evade MX2 restriction is unclear. Currently available data do not support strict correlations between MX2 binding and sensitivity; MX2 binding to CA is reduced by N57S, but not by N74D [299, 300]. MX2 restriction of HIV-1 appears to depend on multiple components of host factors required for nuclear entry [124, 196, 298]. However, CPSF6 depletion did not have noticeable effects on MX2 restriction of HIV-1 [124, 170].

The CA-CTD also affects MX2 sensitivity of HIV-1. A CA mutant library was used to identify two MX2-resistant mutations in the CA-CTD (M185I and E187V) [294]. Additionally, escape mutations in the CA-CTD (P207S, G208R, and T210K) arose under pressure from MX2 inhibition during in vitro evolution experiments [294]. Amino acid residues at these three positions are variable among circulating HIV-1 strains. Such naturally occurring variations contribute to differences in MX2 sensitivity. For instance, threonine is frequently found at position 207 among CRF01_AE variants, but replacement of threonine with a more common proline residue increased MX2 sensitivity [307]. Additionally, MX2 resistance of two transmitted founder variants was shown to be determined in part by the G208A substitution [304, 308]. Natural polymorphism at position 210 also affects MX2 sensitivity; viruses bearing a threonine at position 210 are sensitive to MX2 restriction, whereas those with a serine escape from inhibition by MX2 [170]. These mutations are clustered within a trimeric interface in the mature capsid structure. Interestingly, capsid binding to MX2 seems to be the main mechanism for resistance by these CA-CTD mutations. Specifically, both G208R and T210K mutations reduce MX2 binding to CA complexes [303, 309], supporting the idea that the tri-hexamer interface is a key binding site of MX2 [303].

These studies have revealed that HIV-1 can escape from MX2 restriction by multiple mechanisms. Notably, the three exposed sequence elements described above (β-hairpin, CypA-binding loop, and helix 6) are known to influence HIV-1 restriction by TRIM5α, suggesting a common recognition mechanism of these two groups of restriction factors [261, 294]. Aside from human MX2, other primate and non-primate species encode MX2 proteins with antiviral activity against HIV-1 [294, 306, 310–312]. Divergent lentiviruses differ widely in their sensitivity to MX2 [124, 156, 157, 292, 294, 300, 311, 313]. Further studies need to be done to fully understand how different capsids adapt to the selection imposed by MX2. It should be also noted that the consequence of MX2 binding to the capsid is likely more complicated than currently appreciated, as it was shown that MX2 can not only block HIV-1 infection but can also enhance it, depending on its CA sequences [124].

### HIV-1 capsid inhibitors

A number of small-molecule compounds that bind the HIV-1 capsid have been developed and explored as a new line of antiviral therapy [12, 13]. The HIV-1 capsid is an attractive target, as CA plays diverse roles during HIV-1 replication [5] and displays extreme genetic fragility [24]. Previous investigations of capsid-targeting inhibitors have elucidated their binding mode and provided insights into the mechanism of action and drug resistance profile. The best characterized example of capsid inhibitors is PF74, which binds the capsid at a preformed pocket [314]. This PF74 binding pocket is also targeted by other capsid inhibitors, including BI-1 [225], GS-CA1 [315] and GS-6207 [316]. It is worth mentioning that this group of antivirals do exhibit clear differences in mechanisms of action; PF74 and BI-1 mostly target mature particles, whereas GS compounds can act on both immature and mature particles. Below, we will describe recent advances in our understanding of drug resistance mutations in CA and their phenotypic consequences with emphasis on antiviral drugs targeting the CPSF6 binding pockets.

PF74 binds to the viral capsid at a preformed pocket that is composed of the NTD of one CA subunit and the CTD of another in the CA hexamer [188, 189]. As such, PF74 binds to the CA hexamer with higher affinity than to the CA-NTD [188, 189]. Importantly, PF74 shares its binding site with multiple capsid-binding host proteins, such as CPSF6, NUP153 and Sec24c [95, 187–190]. The EC_50_ of PF74 against HIV-1_NL4-3_ in MT-2 cells was reported to be ∼500 nM [223]. PF74 inhibits HIV-1 infection at various steps, including reverse transcription, nuclear entry, and integration [72, 188, 314, 317–321]. A distinct group of compounds (BI-1 and BI-2) also binds to the same binding pocket [188, 225], though they only interact with the CA-NTD. Recently, a series of capsid inhibitors that target this NTD-CTD interface with much improved potency and efficacy (GS-CA1 and GS-6207) have been developed [315, 316]. The EC_50_ of GS-CA1 against HIV-1_IIIB_ in MT-4 cells is ~ 240 pM, indicating that GS-CA1 has over 2,000-fold higher potency than PF74. This group of compounds that bind the capsid at the same region is currently being evaluated in vivo as a novel agent for HIV-1 treatment [315, 316] but has also served as an important research tool to dissect capsid-mediated processes required for HIV-1 replication. Additionally, comprehensive characterization of drug resistant variants has informed how sequence changes in CA that emerged under selective drug pressure modulate virus-host interactions and viral replicative capacity.

The structure of the PF74-CA complex has been determined [188, 189, 314]. Several amino acid changes in the PF74 binding pocket have been shown to affect PF74 binding to CA and the sensitivity of HIV-1 to this compound. For instance, the N57A mutation in CA abolished PF74 binding to CA and conferred nearly complete PF74 resistance [95, 187, 320]. N74D is another mutation in this pocket that diminished CA binding to the drug [187] and rendered HIV-1 less sensitive to PF74 [320]. Other mutations in the PF74 binding pocket, such as M66F, K70A, and R173A, reduce PF74-CA binding to varying degrees [187, 189].

In vitro serial passage experiments with PF74 or its analogue were performed to identify resistance mutants. The first identified variant is called 5Mut, which carries five amino acid substitutions in CA: Q67H, K70R, H87P, T107N and L111I [223]. 5Mut displays reduced binding to PF74 and confers substantial resistance to PF74 [189, 223, 317]. Three mutations (Q67H, K70R, and T107N) in the drug binding pocket contribute to reduced capsid binding to PF74 [189, 223, 317]. Each of these mutations causes partial PF74 resistance [322]. The 5Mut virus shows a delayed replication kinetics, which is more profound in primary macrophages and T cells [198, 322]. The 5Mut virus differs from the WT virus at least in two properties, which may be the basis for the replication block. First, the 5Mut virus lost the ability to interact with CPSF6 and CypA and became less dependent on NUP153 and NUP358 for infection [198]. Second, cell-free assays showed that these five mutations increase intrinsic capsid stability [192, 317]. Consistent with this observation, 5Mut exhibited a phenotype of stable cores in virus-infected cells in two different assays [71, 317], although this change in capsid disassembly was not observed in another assay [319].

Another PF74 resistant variant was obtained after a long-term serial passage experiment and designated as 4Mut [198]. The 4Mut variant harbors a Q67H change that is also present in the 5Mut variant and reduces binding to PF74 [198]. Other mutations in the 4Mut variant are S41A in the CA-NTD and V165I and L172I in the CA-CTD [198]. This observation fits with the idea that PF74 targets the NTD-CTD interface [188, 189]. Replication of 4Mut is also profoundly impaired in primary cells [198]. The amino acid changes in the 4Mut variant block CA interactions with CPSF6 and NUP153 and reduced the dependence on HIV-1 nuclear entry cofactors [198]. Thus, 5Mut and 4Mut share some common properties.

These two PF74 resistant variants reduce capsid binding to both drug and host cofactors [198, 322]. Thus, it is unclear whether the reduced binding to cofactors has any role in drug resistance. This idea was supported by the observation that mutations that reduce capsid binding to host proteins, such as CypA and CPSF6, decrease PF74 sensitivity of HIV-1. Namely, both CPSF6-binding and CypA-binding deficient mutants were shown to be less sensitive to antiviral activity of PF74 [317, 320]. Furthermore, the K182A mutation, which reduces CA binding to CPSF6, confers partial resistant to low doses of PF74, even though the same mutation does not affect PF74 binding to the CA hexamer [320]. Thus, PF74 can competitively block CA-CPSF6 binding [187], but the lack of CA-CPSF6 binding did not enhance PF74 potency and it even reduces the antiviral activity against HIV-1. These host proteins can change the kinetics of uncoating. The altered uncoating kinetics may serve as one mechanism of resistance, as many of the capsid stability mutants confer partial resistance to PF74 [317, 323]. For instance, the hyper-stable mutant E45A binds to PF74 at a WT-level but is less sensitive to PF74 than the WT virus [317].

GS-CA1 and GS-6207 bind to the capsid at the same site as PF74 [315, 316, 324]. These compounds have been explored as a highly potent long-acting compounds in clinical trials [325]. Resistance-associated mutations for GS-CA1 were identified within the drug binding pocket at the following positions: L56, N57, M66, Q67, K70, N74 and T107 [315]. Serial passage of HIV-1 in the presence of GS-6207 selected for Q67H and N74D substitutions [316]. Additional selection experiments identified resistance mutations at the positions shared by those resistant to GS-CA1 [316]. These include L56I, M66I, K70N, Q67H/N74S and Q67H/T107N mutations [316]. M66I is a key change to confer drug resistance [326] and was shown to counteract inhibitory effects of these compounds on HIV-1 release and endogenous reverse transcription [97, 316].

Amino acid residues involved in resistance to these compounds exhibit a high sequence conservation among major HIV-1 clades. In addition, none of the resistant mutations for GS-6207 were identified in a study investigating plasma samples from 1500 patients infected with HIV-1 [327]. Consistent with these observations, all these mutations resistant to GS-CA1 and GS-6207, except for one, impair viral replicative capacity in both immortalized and primary T cells [315, 316]. The degree of resistance appears to positively correlate with the magnitude of mutational effect on viral fitness. The M66I change conferred the highest resistance to GS-6207 but also drastically decreased viral infectivity. N57S and K70A mutants with an intermediate level of resistance suffered from a considerable loss of infectivity [316]. In contrast, the Q67H substitution confers low-level resistance to GS-6207 and retains WT fitness [316]. Critically, this Q67H replacement was observed in one HIV-1 infected humanized animal treated with GS-CA1 [315]. Furthermore, the same Q67H mutation emerged in one HIV-1-infected patient after a single administration of 20 mg of GS-6207 [316]. It will be critical to continuously monitor how virus with the low-level resistance will evolve in a longer time scale.

Resistance selection studies for BI compounds of which binding site overlaps with those for PF74 and GS-6207 identified substitutions at A105 and T107 residues (A105T and T107A/N) [225]. Note that T107N was also selected for after in vitro adaptation against PF74 and GS-6207. Similarly, mutations (N57A and N57S) that reside in the drug binding pocket also enabled HIV-1 to escape from antiviral activity of BI-2 [197]. Overall, these observations support the idea that HIV-1 gains resistance to this group of compounds (GS-6207, PF74 and BI-2) through common mutational pathways. It is notable that amino acid changes at positions 105 and 107 in CA were selected in multiple occasions (see above). Mutations in these positions tend to reduce capsid binding to CPSF6. Thus, we speculate that the requirement for preserving CPSF6 binding underlies the observation that amino acid changes in this drug binding pocket are rare in vivo. Importantly, recent work showed that a transmitted founder variant is resistant to high doses of PF74 through a mechanism distinct from reduced PF74 binding [90]. Additionally, two SIVcpz strains displayed less sensitivity to PF74 and GS-CA1 [328]. Thus, additional studies of diverse HIV-1 and other lentiviruses may further uncover distinct viral strategies to evade inhibition by these capsid inhibitors.

Benzodiazepines and benzimidazoles belong to another group of capsid inhibitors that binds to the CA-NTD [329]. These compounds either block virus particle production or capsid maturation by interfering with the formation of the NTD/CTD contacts that are critical for stabilizing CA hexamers and pentamers. Resistant mutations were found at the CA-NTD that are close to the drug binding site as well as in the CA-CTD [329]. Interestingly, these CA-CTD mutations, such as G208R and E213G, were often identified together with binding mutations in the CA-NTD. These CA-CTD mutations reside at the trimeric interfaces and were shown to promote CA-NC assembly in cell-free assays, suggesting the possibility that indirect resistant mutations likely modulate the stability of the capsid lattice [329].

Maturation inhibitors (MIs), such as bevirimat (BVM), bind immature virus particles and block HIV-1 infection by interfering with Gag cleavage at the CA-SP1 junction [330] and thus preventing mature capsid formation. While MIs are not classified as capsid-targeting inhibitors, their resistance can be mediated by a few distinct groups of amino acid changes in the CA domain [314, 331–338]. Two groups of resistant mutations are notable. One group contains mutations at the CA-SP1 boundary region. Mutations at both G225 and H226 residues impair virus fitness [332, 336, 339]. In contrast, a V230I substitution, which occurred in a resistant variant after in vitro evolution in the presence of BVM [335], is also present in circulating HIV-1 variants [340]. Thus, sequence variability in CA among primary strains can be a determinant for the sensitivity to HIV-1 inhibitors.

A second region that harbors MI resistant mutations is the MHR in the CA-CTD [336, 337]. These mutations were discovered using a structurally distinct MI called PF96 or second-generation MIs [336, 337]. Consistent with the critical role played by the MHR during virus particle production, most of the resistant mutations in the MHR attenuated viral replication [336]. Mechanistically, two of the mutants, G156E and P157S, were shown to have defects in Gag multimerization [48, 336]. These amino acid residues are located close to K157, which is one of the key amino acid residues responsible for recruiting IP6 in immature virus particles. In fact, recent work revealed an intriguing link between IP6 and MIs; IP6-deficient mutants were shown to be resistant to MIs [47] and rescued by a T-to-I substitution at position 8 of SP1, which has a similar effect to MIs [48]. Unexpectedly, one MI resistant mutation (P157A) in the MHR remains replication-competent even in primary cells [337]. Alanine at position 157 is observed in some SIV strains. Thus, some amino acid residues in the MHR may be tolerable to mutational effects without fitness loss, an observation that is relevant to further consideration of drug resistant mutations in CA.

## Conclusions

This review article has summarized recent findings about how sequence variation in CA causes a remarkable range of variation in phenotypic properties of HIV-1. Capsid interaction with host factors is a key feature that defines the replication strategy of HIV-1. Numerous amino acid changes in CA perturb capsid–host interactions and consequently change various viral phenotypes, including a profound defect in infectivity. Previous work uncovered multiple sites of vulnerability in the viral capsid, some of which are currently being explored as a target for therapeutic interventions. On the other hand, there are several cases in which the loss of host factor utilization by HIV-1 (e.g., CypA, and CPSF6) has negligible impacts on viral fitness, even though it can alter the timing and location of capsid disassembly, change nuclear entry pathways, and also diminish the unique preference of HIV-1 integration targeting. These observations are in accord with other studies indicating that the requirement of these host factors is not conserved among diverse lentiviruses. Thus, these results underscore a striking flexibility in various post-entry processes mediated by the viral capsid. Overall, these findings highlight an unexpected duality of the viral capsid: extreme genetic fragility and functional flexibility.

Another lesson learned from previous work is that a single amino acid replacement in CA can result in multiple, and sometimes independent phenotypic outcomes (Table 1). For instance, the N74 residue is critical for CA binding to CPSF6 [93]. As such, the N74D mutation leads to phenotypes similar to those caused by the loss of CA binding to CPSF6, such as provirus integration into gene-poor regions [105, 214]. The same amino acid change also renders HIV-1 sensitive to antiviral activity of TRIM34, but this differential sensitivity appears to be independent from CA interactions CPSF6 interactions [219]. Another notable example is a conserved arginine residue at position 18, which has been implicated in nucleotide transport and binding to IP6 as well as FEZ1 [45, 96, 101, 102]. These examples highlight the difficulty in attributing an infectivity defect to a single mutational effect, and thus underscore a need for a combinatory approach to define the role of each amino acid of CA in diverse functions of the HIV-1 capsid.

Several questions remain unresolved. In addition to well-characterized CPSF6 and CypA, many other cellular proteins interact with the viral capsid, but their binding modes and utilization by various mutants or other groups of lentiviruses have been less well investigated (e.g., BICD2, FEZ1, and TRN-1) [101, 127, 341–343]. Our understanding of capsid-dependent restriction is incomplete. Previous work identified several different mechanisms of capsid-dependent post-entry blocks that are independent from TRIM5 and MX2 [312, 344–347]. Interestingly, some of such restriction can be attenuated or sensitized by amino acid changes in CA regions that mediate CypA or CPSF6 binding [138, 312, 347, 348]. For instance, antiviral activity elicited by type I IFN responses was shown to be more potent against certain HIV-1 variants carrying CA mutations [138]. In addition, human blood cells express a capsid-specific factor that blocks viruses belonging to the SIVsm/mac lineage [346], while non-human primate and non-primate species encode unknown restriction factors that inhibit HIV-1 in a CA-dependent manner [312, 347]. Finally, lentivirus restriction that is dependent on both envelope and capsid has been described [344, 345] in which one type of restriction, called Lv2 [344], is determined by N74D, G89V, and G94D mutations [348]. Continued investigation of these various types of unresolved capsid-dependent restriction will allow us to better define the exact composition of the antiviral defense system in the host that likely played an important role for shaping the evolution of lentiviruses.

Another challenge relates to the viral genetic background. An increasing number of studies have characterized circulating HIV-1 variants, but most work relies on a handful of lab-adapted HIV-1 variants. Previous work showed that even closely related HIV-1 variants (LAI vs NL4-3) differ in their mutational effects [220]. In other instances, single amino acid changes result in different phenotypic outcomes depending on the viral sequence background [170, 221, 339]. HIV-1 genetic context matters for viral evolution, as the viral sequence background influences how HIV-1 adapts to cellular immunity in vivo [349]. Deep scanning mutagenesis, a technique to dissect mutational effects of each residue in host or viral proteins [350, 351], has been applied to studying the mutational tolerance of the HIV-1 genome and the evolutionary process during HIV-1 adaptation to antiviral agents [352–355]. Future studies that combine such technique with the utilization of different genetic backgrounds should provide new insights into epistatic interactions in the viral capsid and enhance our understanding of the evolutional potential of the HIV-1 capsid.

## Data Availability

Not applicable.

## Abbreviations

BIV: Bovine immunodeficiency virus
cGAS: Cyclic GMP-AMP synthase
CM: Cynomolgus macaque
CPSF6: Cleavage and polyadenylation specificity factor subunit 6
CsA: Cyclosporine A
CTD: C-terminal domain
CypA: Cyclophilin A
EC_50_: Effective concentration 50
EIAV: Equine infectious anemia virus
FIV: Ferine immunodeficiency virus
HIV: Human immunodeficiency virus
IFN: Interferon
IP6: Inositol hexakisphosphate
IPMK: Inositol-polyphosphate multikinase
IPPK: Inositol-pentakisphosphate 2-kinase
MHR: Major homology region
MX2: MX Dynamin like GTPase 2
N-MLV: N-tropic murine leukemia virus
NTD: N-terminal domain
NPC: Nuclear pore complex
PIC: Pre-integration complex
RM: Rhesus macaque
RSV: Rous sarcoma virus
SIV: Simian immunodeficiency virus
TRN-1: Transportin-1
TRIM5: Tripartite motif containing 5
